# The KDM2B- Let-7b -EZH2 Axis in Myelodysplastic Syndromes as a Target for Combined Epigenetic Therapy

**DOI:** 10.1371/journal.pone.0107817

**Published:** 2014-09-16

**Authors:** Ekapun Karoopongse, Cecilia Yeung, John Byon, Aravind Ramakrishnan, Zaneta J. Holman, Peter Y. Z. Jiang, Qiang Yu, H. Joachim Deeg, A. Mario Marcondes

**Affiliations:** 1 Clinical Research Division, Fred Hutchinson Cancer Research Center, Seattle, Washington, United States of America; 2 Department of Anatomic Pathology, University of Washington, Seattle, Washington, United States of America; 3 Department of Hematology, University of Washington, Seattle, Washington, United States of America; 4 Department of Medicine, University of Washington, Seattle, Washington, United States of America; 5 Medical Oncology, Providence Regional Cancer Partnership and the Everett Clinic, Everett, Washington, United States of America; 6 Cancer Biology and Pharmacology, Genome Institute of Singapore, A*STAR (Agency for Science, Technology and Research), Biopolis, Singapore, China; University of Navarra, Spain

## Abstract

Both DNA and histone methylation are dysregulated in the myelodysplastic syndromes (MDS). Based on preliminary data we hypothesized that dysregulated interactions of KDM2B, let-7b and EZH2 signals lead to an aberrant epigenetic landscape. Gene expression in CD34+ cells from MDS marrows was analyzed by NanoString miR array and validated by real-time polymerase chain reaction (PCR). The functions of KDM2B, let-7b and EZH2 were characterized in myeloid cell lines and in primary MDS cells. Let-7b levels were significantly higher, and KDM2B and EZH2 expression was lower in primary CD34+ MDS marrow cells (n = 44) than in healthy controls (n = 21; p<0.013, and p<0.0001, respectively). Overexpression of let-7b reduced EZH2 and KDM2B protein levels, and decreased cells in S-phase while increasing G0/G1 cells (p = 0.0005), accompanied by decreased H3K27me3 and cyclin D1. Silencing of KDM2B increased let-7b expression. Treatment with the cyclopentanyl analog of 3-deazaadenosine, DZNep, combined with the DNA hypomethylating agent 5-azacitidine, decreased levels of EZH2, suppressed methylation of di- and tri-methylated H3K27, and increased p16 expression, associated with cell proliferation. Thus, KDM2B, via let-7b/EZH2, promotes transcriptional repression. DZNep bypassed the inhibitory KDM2B/let-7b/EZH2 axis by preventing H3K27 methylation and reducing cell proliferation. DZNep might be able to enhance the therapeutic effects of DNA hypomethylating agents such as 5-azacitidine, currently considered standard therapy for patients with MDS.

## Introduction

Resistance to spontaneous and therapy-induced apoptosis of clonal hematopoietic cells is central to the progression of myelodysplastic syndrome (MDS) to acute myeloid leukemia (AML). Multiple signals are involved in the dysregulation of hematopoiesis and evolution of MDS [1]–[3]. Major components include altered methylation status of double stranded DNA and histones. The histone(lysine) *demethylase* KDM2B, the first identified human paralog of the Jumanji C (JmjC)-domain-containing histone demethylase 1b (Jhdm1b), has been implicated in cell-cycle regulation and tumorogenesis [4]. KDM2B represses Ink4/Arf via EZH2, the catalytic subunit of the polycomb repressive complex 2, a highly conserved histone *methyltransferase* that targets lysine-27 of histone H3, and interferes with cell proliferation and cellular senescence [5]. Data from murine models suggest that suppression of KDM2B in leukemia stem cells blocks the development of frank leukemia [6]. Other studies indicate that KDM2B transcriptionally represses microRNAs (miRs) such as let-7b [7], which, in turn, target EZH2 expression. EZH2 performs a critical role in epigenetic regulation as a bridge between DNA methylation and other epigenetic modifications such as histone acetylation and methylation [8], [9].

We showed previously a direct interaction between miR10a/b, and another dysregulated transcription factor, TWIST1, in patients with MDS, emphasizing the regulatory role of miRs in the pathophysiology of MDS [10]. This observation is of interest in the light of recent data on the effect of the polycomb repressive complex on H3K27 tri-methylation and TWIST1 expression [11]. We now show that KDM2B promotes transcriptional repression via let-7b and EZH2. Results indicated, further, that the novel cyclopentanyl analog of 3-deazaadenosine, DZNep, bypassed the inhibitory KDM2B/let-7b/EZH2 axis by preventing H3K27 di- and tri-methylation and reducing cell proliferation, suggesting potential usefulness in the treatment of MDS.

## Methods

### Cell lines and marrow samples

KG1 and KG1a cells were obtained from American Type Culture Collection (ATCC) (Rockville, MD). KG1 cells were derived from a patient with relapsed erythroleukemia, showing the phenotype and function typical for myeloblasts. KG1a cells were derived as a subclone from KG1 cells, showing more immature characteristics than KG1 cells. PL-21 cells and ML-1 cells were also purchased from ATCC. PL-21 cells were derived from a patient diagnosed as acute promyelocytic leukemia; they lack t(15;17) and have features of monocytic cells. ML-1 cells were isolated from a patient with acute myeloblastic leukaemia. MDS-L cells were a gift from Prof. Kaoru Tohyama (Hamamatsu University School of Medicine, Japan). This cell line was established as a blastic subline from MDS92 [12]. KG1, KG1a, and ML1 cells were cultured in RPMI with 10% heat-inactivated fetal bovine serum (FBS), 1% sodium pyruvate (Invitrogen, Carlsbad, CA.) and 1% Penicillin/streptomycin (P/S). PL-21 cells were cultured in RPMI plus 20% FBS, 1% sodium pyruvate and 1% P/S. MDS-L cells and CD34+ cells from the marrows of healthy donors or patients with MDS were cultured in Stemspan (STEMCELL Technologies Inc., Vancouver, BC) plus 100 ng/ml of IL-3 (Peprotech, Rocky Hill, NJ, USA). All cell lines were maintained at 37°C in a 5% CO_2_ atmosphere.

Mononuclear cells were separated from fresh marrow aspirates by Lymphocyte Separation Medium (mediatech Inc., Herndon, VA), according to the manufacturer's instruction. CD34+ cells were purified by magnetic bead sorting (Miltenyi Biotec, Auburn, CA), following the manufacture's protocol. Total cell derived RNA or synthetic miR pools were used as input of nCounter miR sample reparation reactions. The analysis of the array data was performed as described before [10].

All research performed in this study was approved by the Fred Hutchinson Cancer Research Center (FHCRC) Institutional Review Board. Written informed consent was obtained from all healthy donors and patients, and all research was conducted within the principles expressed by the Declaration of Helsinki. Marrow and blood cells for studies were obtained under FHCRC protocols 1713 and 208.4144.

### Reagents

DZNep (3-deazaneplanocin A), an inhibitor of histone methyl transferase was provided by Dr. Qiang Yu, Cancer Biology and Pharmacology, Genome Institute of Singapore, A*STAR (Agency for Science, Technology and Research, Biopolis, Singapore). 5-azacitidine (5AZA) was purchased from Sigma (St. Louis, MO) and was used at concentrations of 1 µM to 5 µM, and DZNep at 0.5 µM to 1 µM. Drug-exposed cells were analyzed for apoptosis/viability at various time intervals following exposure.

### Western blot analysis

Total cell lysates and nuclear extracts were assayed using the Nuclear Extraction Kit, according to the manufacturer's instructions (Panomics, Fremont, CA). Fractions were cleared by centrifugation at 13,000 g for 10 minutes. Protein concentrations were quantified by bicinchoninic acid assay (Pierce Biotechnology Inc), and equal amounts of protein (50 µg) from each lysate were diluted in Laemmli sodium dodecyl sulfate sample buffer and resolved by electropheresis on 4% to 12% Bis-Tris precast NuPage gels (Invitrogen, Carlsbad, CA) in running buffer (50 mM 2-(N-morpholino) ethane sulfonic acid, 50 mM Tris [tris(hydroxymethyl)aminomethane]) base, 0.1% sodium dodecylsulfate, and 1 mM EDTA [ethylenediaminetetraacetic acid]). For immunoblotting the proteins were transferred to polyvinylidene difluoride membranes. The membranes were blocked with 5% nonfat dry milk diluted in Tris-buffered saline containing antibodies for the following target proteins: KDM2B (09-864, Millipore, Billerica, MA, USA), EZH2 (612666, BD biosciences), H3K27me2(Lys27) (07-421, Millipore), H3K27me3(Lys27) (07-449, Millipore, Billerica, MA, USA), p16 (Anti-p16, clone D25, Millipore, Billerica, MA, USA) and phospho-cyclin D1(T286, D29B3, Cell signaling, Danvers, MA, USA).

### Real time PCR

Total RNA was isolated from cell lines using the RNAeasy Minikit (Qiagen, Foster City, CA). cDNA for mRNA and miRNA was synthesized from total RNA by using the enzyme RMV from a reverse transcriptase kit (Invitrogen, Carlsbad, CA.) and MicroRNA reverse transcription kit (Applied biosystems, Grand island, NY). Levels of EZH2 (Hs01016789_m1), KDM2B(Hs00404800_m1) and let-7b (assay ID, 2619, Applied Biosystems) expression were determined (in biological triplicates) using Taqman and PCR Master mix (Applied Biosystems, Foster City, CA) on ABI 7500 Real time PCR system (Applied Biosystems, Foster City, CA) for 40 cycles. B-actin and U6B served as internal control for mRNA and miRNA, respectively. The levels of mRNA and miRNA in each sample were normalized to the control and recorded as a relative expression level.

### Overexpression and knockdown of KDM2B and miR let-7b

#### KDM2B plasmid constructs

The retroviral constructs were derived from the vector MigR1, a variant of MigR1 kid, a kind gift of Dr. Alexandros Tzatsos (Massachusetts General Hospital Cancer Center, Boston, USA). This construct was designed to overexpress wild-type and mutant forms of KDM2B [7]. For conditional inhibition of KDM2B the doxycycline-inducible lentiviral construct pTRIPZ (Openbiosystems, Huntsville, AL, USA) was used.

#### Lentiviral vectors

mZ-CTRL (miRZip-control) and miR let-7b were obtained from System Biosciences (Mountain View, CA, USA). The let-7b miRZip delivers short anti-sense RNAs that are stably expressed and competitively bind their respective endogenous miR targets, thereby inhibiting their function.

### Proliferation and apoptosis

Cells were plated in triplicates in 24-well plates (2×10^5^ cells/well). Apoptosis was assessed by flow cytometry using Annexin V–FITC labeling. Proliferation and cell cycle phase were determined using the APC-BrdU Flow Kit (BD Biosciences, Mountainview, CA, USA). Cell cycle analyses were performed using a FACSCalibur (BD Biosciences, Valencia, CA, USA) with CellQuest Pro software provided by the manufacturer.

### Immunohistochemistry

Formalin-fixed paraffin embedded marrow particle blocks were sectioned at a thickness of 4 µm and dried at room temperature. The sections were deparrafinized using a standard xylene/ethanol protocol before antigen retrieval according to a Dako (Dako, Carpenteria, CA, USA) protocol [13], using 20 min steam in targeted retrieval solution, pH6.0. The sections were protein blocked with TCT buffer before application of the rabbit monoclonal antibody against EZH2 (cloneM18, D2C9, Cell Signaling Technologies, Danvers, MA) diluted to 1∶300, for one hour. Immunohistochemistry was performed using a DAKO Autostainer (Dako, Carpenteria, CA, USA). The PowerVision Rabbit HRP polymer (Leica, Buffalo Grove, IL, USA) was used as the detection system followed by Dako Dab Plus (Dako, Carpenteria, CA, USA).

Marrow cells and tonsillar tissue from healthy donors were used for method optimization. Concentration matched isotype controls were included in all experiments. Staining was assessed using a semi-quantitative grading system to determine the intensity of staining and the proportion of myeloid cells and blasts showing a positive nuclear reaction.

### Statistical analysis

Statistical significance between two groups was determined using unpaired, two-sided Student's t-test. For multiple group comparisons, statistical significance was determined by one-way analysis of variance (ANOVA) with Tukey's multiple comparison test.

The correlation between two variables was calculated by using Pearson's Correlation Coefficient. The level of significance was set at p≤0.05. All tests were performed using SPSS software (version 18.0; IBM SPSS, Chicago, IL, USA).

## Results

### MiR let-7b, KDM2B, and EZH2 expression in myeloid cell lines and primary MDS marrow cells

**Figure 1:**
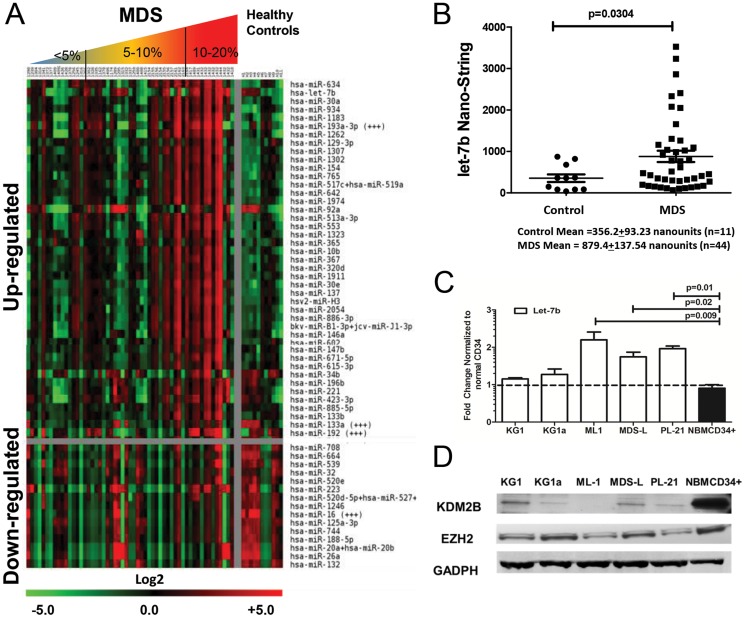
CD34+ marrow cells from MDS patients showing distinct patterns of up-regulated and down-regulated miRs in comparison to healthy controls. **A**) Heatmap of the top 43 up-regulated and the top 15 down-regulated miRs in CD34+ marrow cells from healthy controls (n = 11) and patients with MDS (n = 44) ordered by <5% blast (n = 14), 5–10% (n = 17) and 10–20% blasts (n = 13), as determined by nanostring micro-array analysis (two replicates). Expression levels were calculated by averaging the duplicate spots for each miR. MiRs are listed in order of significance (top up-regulated vs. top down-regulated) determined by SAM analysis, displayed is log 2 value. Higher levels of miRs are displayed in red, lower levels in green. **B**) NanoString Array data of individual samples, shows increased expression of let-7b in MDS (n = 44) compared to CD34+ cells from health controls (n = 11; mean ± SEM = 356.2±93.23 versus 879.4±137.54, p = 0.03 in patients with MDS, Student t-test). **C**) Let-7b expression (by RQ-PCR) in myeloid cell lines ML-1, PL-21, MDS-L, KG1 and KG1a compared to primary marrow cells from a pool of 6 healthy donors (NBMCD34+)(mean ± SEM of 3 experiments, Fold changes, normalized to expression in healthy donors; Student t-test). **D**) KDM2b and EZH2 expression (by western blot) in the same cell lines. The lowest levels of KDM2B and EZH2 are seen in ML-1 and PL-21 cells, the cells with the highest levels of let-7b. GADPH served as control (The blots show one of 3 similar experiments).

In agreement with earlier preliminary studies [10] nano-string array analysis of primary CD34+ MDS marrow cells showed increased expression of let-7b compared to CD34+ marrow cells from healthy donors (p = 0.0304, **Figure 1A,1B**) (also see Supporting Array Data File S1). The observed increase in let-7b levels was further confirmed when miRs array expression was stratified by myeloblast count (**Table 1**). Results in cell lines varied: let-7b expression was higher in ML1, PL-21 and MDS-L than in KG1 and KG1a cells. As studies in other models had suggested that several miRs, including let-7b, were regulated by KDM2B [7], [14], we analyzed the relationship of KDM2B and let-7b in these myeloid cell lines. KG1 and KG1a cells showed constitutively low expression of let-7b, and western blots revealed higher levels of KDM2B than in ML1, MDS-L and PL-21 cells, which expressed high levels of let-7b (**Figure 1C,1D**). This inverse correlation of let-7b and KDM2B was consistent with the concept that KDM2B regulates the expression of let-7b in clonal hematopoietic cells.

**Table 1:** MiRs expression in CD34+ MDS marrow cells ordered by blast count.

Blast Count <5% (N = 14)		Blast Count (5–10%) (N = 17)		Blast Count (10–20%) (N = 13)	
hsa-miR-634	3.47	hsa-miR-634	3.18	hsa-miR-644	6.47
hsa-miR-193a-3p	3.19	hsa-miR-193a-3p	2.96	hsa-let-7b	5.68
hsa-miR-1183	2.78	hsa-let-7b	2.84	hsa-miR-885-3p	4.38
hsa-miR-934	2.59	hsa-miR-92a	2.80	hsa-miR-339-5p	3.59
hsa-miR-1262	2.53	hsa-miR-1183	2.58	hsa-miR-590-5p	3.27
hsa-miR-30e	2.48	hsa-miR-584	2.26	hsa-miR-494	2.91
hsa-miR-518e	2.39	hsa-miR-513a-3p	2.22	hsa-miR-320c	2.90
hsa-miR-30a	2.35	hsa-miR-934	2.19	hsa-miR-196a	2.80
hsa-miR-765	2.32	hsa-miR-30a	2.18	hsa-miR-519d	2.80
hsa-miR-584	2.27	hsa-miR-1262	2.17	hsa-miR-27b	2.72
hsa-miR-1246	−3.18	hsa-miR-561	−2.60	hsa-miR-1975	−4.34
hsa-miR-16	−3.21	hsa-miR-1246	−2.81	hsa-miR-15b	−4.45
hsa-miR-539	−3.28	hsa-miR-16	−2.84	hsa-miR-92a	−4.47
hsa-miR-125a-3p	−3.36	hsa-miR-125a-3p	−2.99	hsa-miR-525-3p	−4.62
hsa-miR-744	−3.36	hsa-miR-744	−2.99	hsa-miR-302c	−4.91
hsa-miR-188-5p	−3.48	hsa-miR-708	−3.14	hsa-miR-423-3p	−4.96
hsa-miR-520d	−3.51	hsa-miR-188-5p	−3.33	hsa-miR-491-5p	−4.99
hsa-miR-132	−3.89	hsa-miR-132	−3.52	hsa-miR-425	−5.02
hsa-miR-26a	−4.26	hsa-miR-26a	−3.89	hsa-miR-450b-5p	−5.31
hsa-miR-20a/b	−4.46	hsa-miR-20a/b	−4.09	hsa-miR-101	−5.37

The top 10 up-regulated and the top 10 down-regulated miRs in CD34+ bone marrow cells from patients with MDS (n = 44) with <5% blasts (n = 14), 5–10% (n = 17) and 10–20% blasts (n = 13) as determined by nanostring micro-array analysis (two replicates), separated displayed are log 2 values. The patterns of miRs up- and down-regulated were nearly identical for MDS cases with <5% and 5–10% myeloblasts, but differed significantly from the pattern in cases with 10–20% myeloblasts. Of note there was some upregulation of let-7b present with 5–10% myeloblasts, but there was even greater upregulation in cases with 10–20% myeloblasts.

Epigenetic alterations induced by KDM2B are linked to H3K27methylation. These changes lead to chromatin condensation and transcriptional repression of let-7b which, in turn, results in increased levels of EZH2 protein [15]. Therefore, we determined next if, conversely, EZH2 levels were affected by expression of let-7b or KDM2B. We reasoned that let-7b expression would post-transcriptionally control EZH2.

In primary CD34+ MDS BM cells (n = 21) expression of KDM2B and EZH2 mRNA was lower than in CD34+ BM cells from healthy donors (n = 11; p = 0.012 and <0.0001, respectively). Let-7b expression was inversely correlated with levels of EZH2 (p = 0.029, R^2^ = 0.1699) and KDM2B (p = 0.051, R^2^ = 0.054), and KDM2B mRNA expression correlated significantly with EZH2 mRNA levels (p = 0.01, R^2^ = 0.2830, 95%CI 0.1300–0.7837).

**Figure 2:**
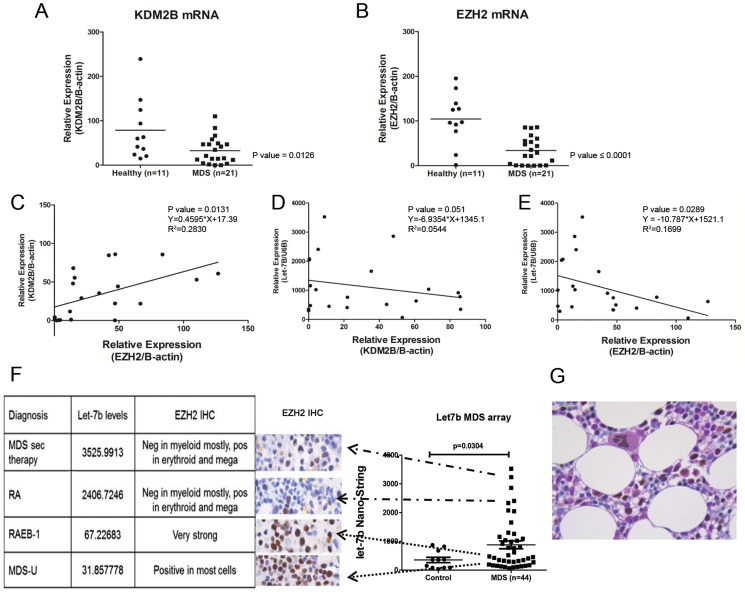
KDM2B, and EZH2 expression in myeloid cell lines and primary MDS marrow cells. Expression of KDM2B, **A**) and EZH2, **B**) in marrow cells from MDS patients (n = 21) and healthy controls (n = 11) (p = 0.0126 and <0.001, respectively, Student t-test). **C**) Direct correlation of KDM2B and EZH2 (p = 0.0131, R^2^ = 0.2830, 95%CI 0.1300–0.7837), **D**) Inverse correlation of let-7b and KDM2B, **E**) Inverse correlation of let-7b and EZH2. **F**) Immunohistochemistry (IHC) for EZH2 on marrow sections from 6 patients with MDS and linkage to Let-7b levels as determined by miRNA microarray. BM CD34+ from MDS patients expressing higher levels of let-7b expressed lower levels of EZH2, and vice versa. **G**) EZH2 immunohistochemistry on a normal marrow with Periodic Acid-Schiff stain as the counter stain was used for the purpose of lineage differentiation. The large cells with brown nuclear staining are a morphologically normal megakaryocytes, positive for EZH2. Of the remaining cells, eosinophilic cells of myeloid lineage and show low level variable reactivity for EZH2, while the remaining erythroid cells show dim to moderate reactivity for EZH2, underscoring the relevance of lineage specific EZH2 expression.

To determine whether mRNA levels were reflected at the protein level, marrow blocks from eight MDS cases (3 in the upper quartile, and 3 in the lower quartile of let-7b levels among 44 patients) were analyzed for EZH2 and KDM2B expression by immunohistochemistry. Marrows from healthy donors showed that erythroid precursors and megakaryocytes expressed EZH2 while the majority of myeloid precursors did not (**Figure 2G**). As shown in **Figure 2F**, in marrows biopsies from MDS patients, myeloid precursors and myeloblasts showed an inverse relationship of EZH2 and let-7b expression. IHC using a polyclonal antibody against KDM2B could not be reliably interpreted due to high levels of non-specific staining (data not shown). Taken together, however, these data support the hypothesis that in clonal hematopoietic cells let-7b post-transcriptionally controls EZH2 expression. Further, and in agreement with other investigators [7], we suggest that the KDM2B/let-7b axis regulates EZH2 levels in clonal hematopoietic cells.

### Modification of KDM2B and Let-7b expression in cell lines

#### Let-7b knockdown and overexpression of KDM2B

**Figure 3:**
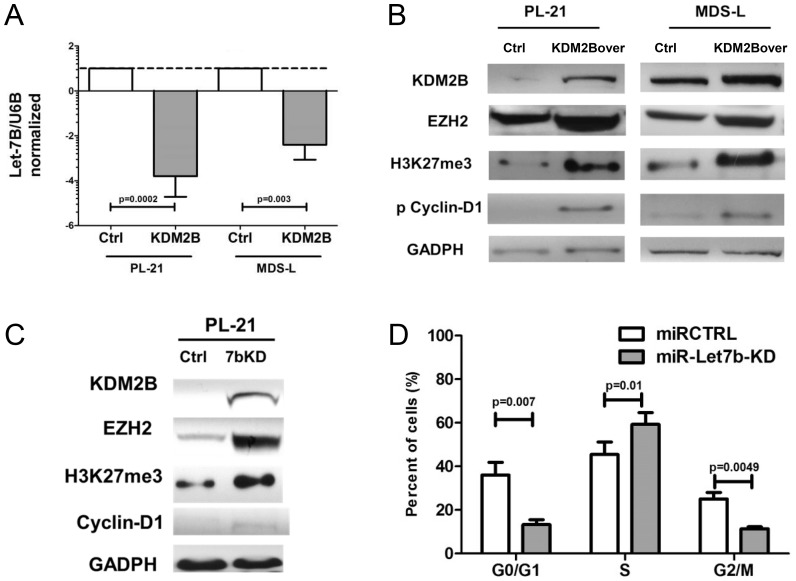
Effects of Let7b knockdown and KDM2B overexpression in myeloid cell lines. Overexpression of KDM2B in PL-21 and MDS-L cells resulted in decreased expression of let-7b (**A**) compared to controls (Ctrl; p = 0.0002 and 0.005, respectively, U6B served as loading control, mean ± SEM, Log 2 changes normalized for the expression in MDS-L cells) and increased EZH2, H3K27me3 and pCyclin-D1 proteins. (**B**) (GADPH served as control). The blots show one of 3 similar experiments. **C**) *Knockdown* of let-7b in PL-21 cells resulted in increased expression of KDM2B, EZH2, H3K27me3 and, to a lesser extent, cyclin-D1. The blots show one of 3 similar experiments. **D**) Knockdown of let-7b in PL-21 cells resulted in increased BrdU uptake/proliferation (increase of cells in S phase; p = 0.01 and a decrease of cells in G1 and G2; p = 0.007 and p = 0.005, respectively; mean±SEM of 3 experiments; Student's t-test for comparison of continuous variables).

To further validate the transcriptional repression of let-7b by KDM2B, we overexpressed KDM2B in MDS-L and PL-21 cells (constitutively low levels of KDM2B). As expected, both cell lines expressed significantly decreased levels of let-7b (p = 0.002 and 0.003, respectively, **Figure 3A**), while western blots showed an increase in EZH2, H3K27me3, as well as phospho-cyclin D1(T286A), a marker of cell proliferation (**Figure 3B**). Similar changes in EZH2, H3K27me3, and phospho-cyclin D1 protein levels were observed following *direct* knockdown of let-7b in PL-21 cells (**Figure 3C**). Of note, knockdown of let-7b also resulted in increased expression of KDM2B – whether EZH2 and KDM2B were affected by let7b knockdown independently from each other remains to be determined. Knockdown experiments were not performed in MDS-L cells, which do not show detectable constitutive levels of let-7b.

Suppression of let-7b by anti-sense mir-Zip let-7b also resulted in increased proportions of cells in S phase(p = 0.01), while fewer cells were present in G1 phase (p = 0.007) (**Figure 3D**).

These experiments indicate that KDM2B can enhance EZH2 expression *via repression of let-7b*. Conversely, interference with let-7b affects EZH2 independently of KDM2B, leading to an altered state of histone methylation and increased cell proliferation.

#### Let-7b overexpression and KDM2B knockdown

**Figure 4:**
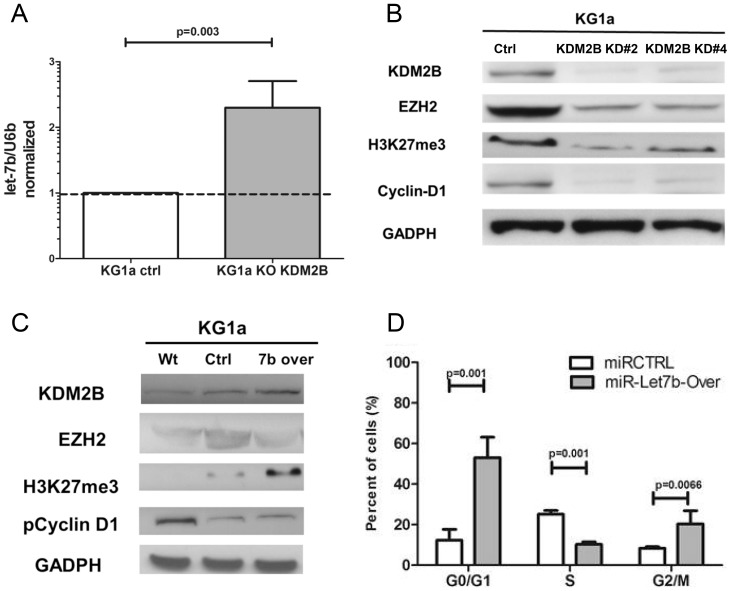
Let-7b overexpression and KDM2B knockdown. **A**) Knockdown of KDM2B in KG1a cells was associated with increased expression of let-7b (p = 0.003, Results show the mean±SEM of 3 experiments; Student's t-test, U6B served as loading control) **B**) Knockdown of KDM2B (constructs #2 and #4) in KG1a cells reduced KDM2B, EZH2, H3K27me3 and Cyclin-D1 protein levels. The blots show one of 3 similar experiments. **C**) KG1a cells overexpressing let-7b showed increased KDM2B and H3K27m3, and decreased EZH2 protein expression. There was no measurable change of pCyclinD1; GADPH served as control. The blots show one of 3 similar experiments. **D**) KG1a cells overexpressing let-7b showed decreased BrdU uptake/proliferation (decrease of cells in S phase; p = 0.001, and increase of cells in G1 and G2, p = 0.001 and p = 0.0066, respectively; results show the mean±SEM of 3 experiments; Student's t-test).

Let-7b expression in CD34+ MDS marrow cells was higher than in healthy controls. Therefore, we overexpressed let-7b, and in parallel experiments silenced KDM2B in KG1a cells (low levels of let-7b and high endogenous levels of KDM2B) with the intent of mimicking the findings in CD34+ MDS cells. Knockdown of KDM2B resulted in increased levels of let-7b as determined by RQ-PCR, while levels of EZH2, H3K27me3 and phospho-cyclin D1(T286A) protein declined. In KG1a cells overexpressing let-7b, levels of EZH2 decreased, while KDM2b and H3K27me3 increased, consistent with the observation reported by Pfau et al. [5]. Further, overexpression of let-7b in KG1a cells led to a reduced proportion of cells in S phase (p = 0.001), and an increase of cells in G1 and G2 phase (p = 0.001, and 0.007, respectively) (**Figure 4**).

These results indicate that KDM2B and let-7b impact histone methylation, apparently via -EZH2 expression, thereby modulating the regulation of cell proliferation involving altered phospho-cyclin D1 expression.

### Response of cell lines and primary marrow cells to DZNep and 5AZA

DZNep selectively inhibits tri-methylation of lysine 27 on histone H3 (H3K27me3), and thereby leads to depletion of the polycomb subunit PRC2 [16]–[18]. On the other hand, 5AZA, used widely to treat patients with MDS, is an inhibitor of the DNA methyltransferase [19]. Thus, both 5AZA and DZNep are involved in epigenetic modulation, but at different levels. We were, therefore, interested in dissecting the effects of these two compounds on let-7b, KDM2B and EZH2, and explored the potential of combined therapy in MDS.

#### Cell lines

**Figure 5:**
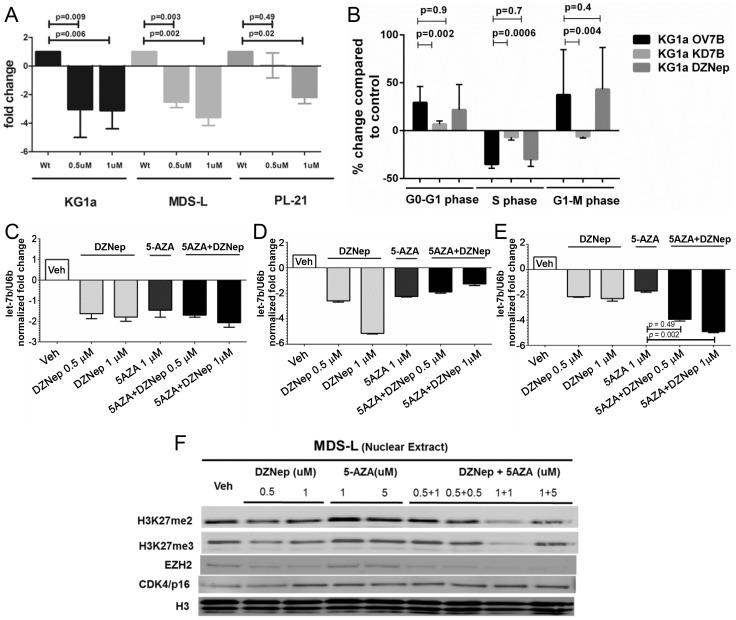
Effects of DZNep and 5AZA on let-7b and EZH2 levels. **A**) DZNep exposure at different concentrations (0.5 µM and 1 µM) significantly reduced let-7b expression in KG1a, MDS-L and P-L21 cells (p = 0.005 and p = 0.002, respectively). The results are displayed as mean ±SEM of 3 similar experiments (Student t-test). **B**) KG1a cells treated with DZNep showed decreased cells in S phase. **C**) Treatment of KG1a cells, **D**) healthy donor cells, and **E**) cells from MDS marrow, with DZNep, 5AZA or a combination of both, decreased let-7b expression, more so with the combination than with 5AZA alone (p = 0.049 and p = 0.002, respectively, results show the mean±SEM of 3 experiments; Student's t-test, U6B served as loading control)); **F**) MDS-L cells showed decreased H3K27m2 and H3K27m3, and decreased EZH2 protein expression.; Histone H3 served as nuclear loading control. The blots show one of 3 similar experiments.

Treatment of KG1a and MDS-L cells with DZNep at doses of 0.5 to 1 µM for 24 hours decreased levels of let-7b as determined by RQ-PCR (**Figure 5A**), associated with a decrease in the proportion of cells in S phase, while cells in G1 phase were increased (**Figure 5B**) (n = 6, p = 0.0008).

Combined treatment with DZNep (0.5–1.0 µM) and 5AZA (1 µM, for 96 hours) administered concurrently, decreased levels of let-7b more profoundly than 5AZA alone in both KG1a cells and primary MDS marrow (**Figure 5C**).

**Figure 6:**
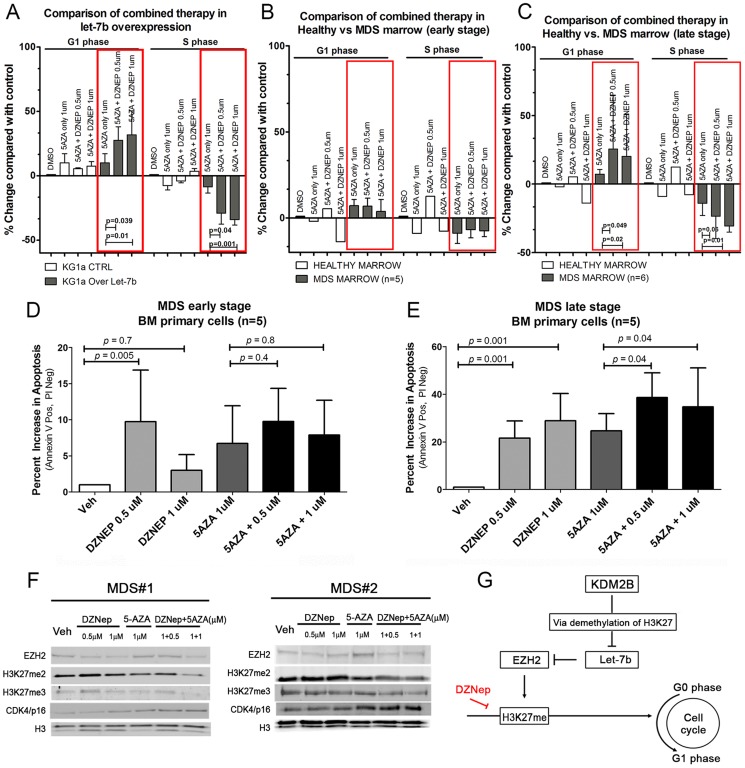
Response of cell lines and primary marrow cells to DZNep and 5AZA. Proliferation study in, **A**) KG1a cells overexpressing let-7b, **B**) primary marrow cells from healthy donors and MDS patients with <10% marrow blasts, and **C**) MDS patients with 10%–20% marrow myeloblasts. Exposure to DZNep, 5AZA or both affected the proliferation in KG1a cells overexpressing let-7b and cells from MDS marrows with 10%–20% but not in cells from patients with <10% marrow blasts (or healthy donors). Exposure to DZNep, 5AZA or combination of both agents (inside of red boxes) affected the proliferation in KG1a cells overexpressing let-7b and cells from MDS marrows with 10–20% but not in cells from patients with <10% marrow blasts or healthy donors. **D**) and **E**) show the extent of apoptosis induced by DZNep, 5AZA or both in MDS patients with <10% and 10%–20% marrow blasts, respectively. The extent of apoptosis was significantly greater in MDS marrows with higher myeloblast counts, more so in cells being treated with the drug combination than with single agents (p = 0.04, 0.001). The results are displayed as mean ±SEM for each MDS group (5 samples for <10% marrow blasts and 6 samples for 10–20% marrow blasts) (Student t-test). **F**) Two different primary BM CD34+ cells specimens showing decreased H3K27m2 and H3K27m3, and decreased EZH2 protein expression upon combined treatment with Dznep+5AZA; Histone H3 served as nuclear loading control. **G**) Working model: role of KDM2B/let-7b/EZH2 in epigenetic regulation of cell proliferation in MDS. KDM2B represses let-7b and up-regulates EZH2. DZnep treatment inhibits histone: H3K27; methylation and bypasses the KDM2B/let-7b/EZH2 effect leading to reduced cell proliferation.

In KG1a cells, overexpression of let-7b (to mimic the findings in CD34+ MDS cells) decreased cells in S phase, and increased the proportion in G1 compared to unmodified KG1a cells. The combination of both compounds had a more profound effect than either drug alone (**Figure 6A**).

Western blot analysis of MDS-L cells treated with a combination of DZNep (0.5 and 1.0 µM) and 5AZA (1 and 5 µM, for 96 hours) revealed reduced levels of H3K27me2 and H3K27me3 associated with decreased levels of EZH2 and increased expression of p16 (**Figure 5F**). These results indicate that KDM2B and let-7b impact histone methylation, apparently via EZH2 expression, thereby modulating the regulation of cell proliferation.

#### Primary marrow cells

We next determined the effects of DZNep and 5AZA in 11 marrow aspirates from patients with newly diagnosed MDS (with <10% myeloblasts, or with ≥10% but less than 20% myeloblasts). Cells derived from patients with ≥10% myeloblasts showed a pronounced decrease in the proportion of cells in S phase along with an increase in G phase (**Figures 6A–6C**). Cells from marrow with lower blast counts showed a significantly lesser response following exposure to DZNep plus 5AZA, which, in fact, did not differ significantly from responses in cells from healthy donors (n = 5, p = 0.7 and p = 0.8, respectively). Cells from patients with >10% myeloblasts showed significantly higher rates of apoptosis in CD34+ cells (presumably clonal) when compared to cells from MDS patients with lower myeloblast counts (and to healthy marrow controls); the effect of the drug combination was more profound than exposure to either DZNep or 5 AZA alone (n = 5, p = 0.04 and p = 0.001, respectively) (**Figure 6E**). In contrast, the rate of apoptosis following drug exposure in cells from patients with lower myeloblast counts did not differ significantly from healthy controls (n = 5, p = 0.4 and p = 0.5, respectively) (**Figure 6D**). Western blot analysis of nuclear extracts from primary MDS marrow cells following combined drug exposure confirmed the pattern found in myeloid cell lines. Decreased levels of H3K27me2 and H3K27me3 were associated with reduced levels of EZH2 and increased levels of p16 in nuclear extracts obtained from the marrow cells of two different MDS patients following exposure to 5AZA at 1 µM and DZNep at 1 µM (**Figure 6F**). Furthermore, the data indicate that exposure to both drugs had a suppressive effect on histone methylation, thereby modulating the regulation of cell proliferation.

## Discussion

Extensive recent work has defined the mutational landscape of MDS [20]–[24], while numerous reports have described aberrant expression of miRs [10], [25]–[27] and transcription factors, even in the absence of mutations [28]–[30]. However, with few exceptions, for example in patients with partial deletion of chromosome 5 (del(5q)), studies on the clinical impact of the molecular findings are limited. Jiang et al. showed a correlation between aberrant DNA methylation and MDS progression [31], and Will et al. reported stage-specific genetic and epigenetic alterations [32]. We observed previously up-regulation of the transcription factor TWIST1 [28] in patients with advanced MDS, and recently showed that TWIST1 controlled miRs10a/b, thereby interfering with apoptotic cell death [10].

In the present study we pursued further the potential role of regulatory interactions of miRs and transcription factors in the epigenetics of MDS. CD34+ MDS marrow cells showed significant dysregulation of EZH2, which was controlled by let-7b. MDS cells overexpressing let-7b responded to treatment with 5AZA plus DZNep with a further decrease of H3K27me2, H3K27me3 and EZH2 expression. In agreement with the findings by others that the histone demethylase KDM2B transcriptionally repressed clusters of miRs including let-7b [7], thereby post-transcriptionally controlling EZH2, our findings show that silencing of KDM2B resulted in increased levels of let-7b (See Working Model, **Figure 6F**). Further, and consistent with that model, up-regulation of let-7b was accompanied by down-regulation of EZH2. As overexpression of KDM2B promoted cell proliferation (increased S-phase entry and G_2_-M transition), down-regulation in response to let-7b would be expected to interfere, as shown, with cell proliferation. These data were further validated by knockdown of KDM2B by siRNA, which inhibited cell proliferation and induced apoptosis. The same effect was observed following pharmacological inhibition with DZNep. Thus, these studies confirm the regulation of transcription and cell cycle progression in MDS at several levels and suggest that DZNep, if clinically tolerated, should be useful as a therapeutic agent in MDS. In fact, the preliminary data generated here indicate that DZNep might enhance the therapeutic efficacy of 5AZA.

No significant effect of DZNep exposure or EZH2 inhibition on cell proliferation or apoptosis was detectable in CD34+ marrow cells from healthy donors, consistent with reports by others [16]. Of note, however, this was also true for marrow cells from patients with low myeloblast percentage and, presumably, early stage MDS. The reason for this is not clear, and several possibilities must be considered. Based on our hypothesis that dysregulation of let-7b occurred only in clonal cells, the simplest explanation would be that the very low myeloblast count in some of these MDS patients did not allow to demonstrate dysregulation of let-7b and that only the expansion of myeloblasts would permit to show differences in comparison to healthy marrow. Alternatively, dysregulated expression may occur only as a feature of the progression of MDS, reminiscent of what we observed with TWIST1 [28], and the transcriptional control of miR 10a/b, possibly in response to signals such as let-7b.

The data indicate that DZNep-mediated suppression of EZH2 activity, as measured by a decrease in the H2K27Me2 and H3K27Me3 histone marks, resulted in inhibition of cell-cycle progression and increased apoptosis, supporting a role of EZH2 in the progression of MDS to a more advanced stage, in conceptual agreement with stage-specific epigenetic alterations in MDS, as reported by others [32]. As observed in solid tumors and immortalized fibroblasts [7], [14]. EZH2 expression in primary MDS cells is maintained by the histone demethylase KDM2B. Results presented here, as well as previous reports [16], [33], [34] indicate that depletion of EZH2, be it by genetic inhibition or by the use of DZNep, interferes with cell-cycle progression through S and G2/M phases and with survival of clonogenic cells by allowing for increased apoptosis. Combined exposure to DZNep and 5AZA resulted in enhanced cytotoxicity against MDS cells, providing a rationale to develop and test this drug combination in patients with MDS. It is of note that EZH2 mutations are observed in 6% of patients with MDS and are associated with worse overall survival compared to patients with wild-type EZH2 [19]. Such an effect of loss of function mutations in EZH2 may not be surprising, as EZH2 maps to chromosome 7, and a loss of 7 is associated with poor prognosis. Among patients included in our analysis, only 5 out of 44 patients had a complex karyotype or monosomy 7 therefore, one could speculate that mutation of EZH2 could respond differently to DZNep. Currently, little is known about specific molecular factors determining DZNep response or resistance. However, emerging data suggest that different tumors are likely to respond to DZNep in a heterogenous fashion [35], [36]—for example, estrogen receptor (ER)-negative breast cancers with low BRCA1 levels may be particularly DZNep-sensitive [35]. Additional experiments are required to test the efficacy of DZNep and the combination with 5AZA in MDS derived cells with EZH2 mutated background.

In summary, the KDM2B/let-7b/EZH2 axis is involved in epigenetic regulation in MDS, with direct effects on di- and tri-methylation of H3K27. KDM2B, via let-7b/EZH2, promotes transcriptional repression in myeloid cell lines and primary MDS cells. This repressive effect is bypassed by DZNep via suppression of PRC2 and H3K27 methylation, leading to reduced cell proliferation and increased apoptosis. These findings provide the rationale to further test the EZH2 antagonist DZNep combined with hypomethylation agents in patients with advanced MDS. Those studies should include determination of let-7b expression as a potential biomarker and predictor of response to combination therapy with DZNep+5AZA.

## Supporting Information

Supporting Array Data File S1 (XLS)
